# Characterization of the complete mitochondrial genome of the Bazhou yak (*Bos Grunniens*)

**DOI:** 10.1080/23802359.2019.1668736

**Published:** 2019-09-25

**Authors:** Chun Huang, Donghai Fu, Xiaoyun Wu, Min Chu, Xiaoming Ma, Congjun Jia, Xian Guo, Pengjia Bao, Ping Yan, Liang Chunnian

**Affiliations:** Key Laboratory for Yak Breeding Engineering of Gansu Province, Lanzhou, People’s Republic of China;; Institute of Husbandry and Pharmaceutical Sciences, Chinese Academy of Agricultural Sciences, Lanzhou, People’s Republic of China

**Keywords:** Bazhou yak, mitochondrial genome, phylogenetic analysis

## Abstract

Yak is an ancient breed and Bazhou Yak is also one of China's unique yak breed resources. In the present study, its complete mitochondrial genome was assembled from Illumina sequencing data and we identified the complete mitochondrial genome of the Bazhou yak (*Bos grunniens*). The complete mitochondrial DNA is a circular molecule with 16,325 bp length consisting of 13 protein-coding genes, 2 rRNA genes, 22 tRNA genes, and a non-coding control region (D-loop). The overall nucleotide composition is A (33.69%), T (27.30%), C (25.79%), and G (13.22%), respectively. The content of C + G is 39.01%. Phylogenetic analysis of the mitochondrial genomes of 15 related species by MEGA7.0 showed that the genetic relationship of Bazhou yak is closer to Datong yak and polled yak.

Yaks are mammals living in the highest altitudes in the world, and China has the largest number of yaks (Wiener et al. 2003). The yak is a treasure that can provide people with quality meat products, dairy products, and plush products (Qiu et al. 2012). Bazhou yak is one of the yak breeds in China, mainly distributed in Mongolian Autonomous Prefecture of Bayingolin, Xinjiang Uygur Autonomous Region. Bazhou yak is a yak group formed by long-term breeding of local Mongolian herders under the specific natural conditions of the production area, adapting to the alpine meadow grassland and alpine grassland environment. The typical characteristics of Bazhou Yak are good meat production traits and strong environmental adaptability. Since mitochondrial DNA is a powerful molecular tool, we have assembled and characterized the whole mitochondrial genome sequence of Bazhou Yak to compare its genetic evolution with other yak breeds in this study (Cameron 2014).

In this work, the blood samples of Bazhou yak were collected from Kuerle county, Mongolian Autonomous Prefecture of Bayingolin, Xinjiang Uygur Autonomous Region (N4145′27″, E86°0844″). These specimens are preserved at −20 °C in the Key Laboratory of Yak Breeding Engineering of Gansu Province, Lanzhou Institute of Husbandry and Pharmaceutical Sciences (Lanzhou, Gansu Province, China) with a storage number: LZ2019-181. Referring to the manufacturer's instructions, total genomic DNA was extracted from blood samples by using Easy Pure Blood Genomic DNA Kit (Transgen Biotch, Beijing, China). We amplified the whole mitochondrial genome with 6 pairs of primers by polymerase chain reaction method and assembled the sequencing results using DNASTAR5.0 software (Madison, WI, USA). The complete mitochondrial genome of Bazhou yak was determined and deposited in GenBank with an accession number: MN175233.

The entire mitochondrial DNA is a circular DNA molecule with a total length of 16,325 bp, which contains the base composition of 33.69% for A, 27.30% for T, 25.79% for C and 13.22% for G, and the content of C + G is 39.01%. Furthermore, this mitochondrial DNA contains the typical structure including a non-coding control region (D-loop), 22 transfer RNA genes,2 ribosomal RNA subunit genes (12s and 16 s rRNA) and 13 protein-coding genes (ATP6, COX1-3, CYTB, ND4L, ND1-6, and ATP8). The two rRNAs are 957 bp (12S rRNA) and 1571 bp (16S rRNA) in length, respectively, and are separated by tRNA-Val whose length is 67 bp. We found three overlaps in the protein-coding genes of this sequence, including ND4 overlaps with ND4L for 7 bp, ATP6 overlaps with COX3 for 1 bp and ND5 overlaps with ND6 for 17 bp. The alignment of these genes is conservative compared to other yak subspecies and Bovidae.

Based on 15 complete bovine mitochondrial genomes, we constructed a phylogenetic tree using MEGA7.0 software combined with the neighbor-joining (NJ) method of 1000 bootstrap replicates (Cummings 2004). The molecular phylogenetic analysis revealed that 15 species are divided into two branches as a whole and Bazhou yak has a closer genetic relationship with Datong yak and polled yak (Figure 1). The sequence analysis provided in this study will be helpful to the management of yak breeds, the origin, and evolution of yak, and the protection and utilization of genetic resources.

**Figure 1.**
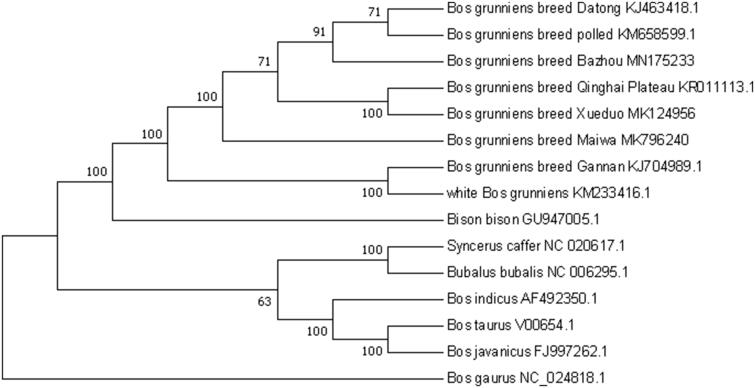
Phylogenetic relationships of mitochondrial genomes of 15 species based on the neighbor-joining (NJ) methods. The result was validated by 1000 bootstraps and the bootstrap values are shown next to the branches.
